# Negative dream affect is associated with next-day affect level, but not with affect reactivity or affect regulation

**DOI:** 10.3389/fnbeh.2022.981289

**Published:** 2022-10-19

**Authors:** Pilleriin Sikka, Hilda Engelbrektsson, Jinxiao Zhang, James J. Gross

**Affiliations:** ^1^Department of Psychology, Stanford University, Stanford, CA, United States; ^2^Department of Psychology, University of Turku, Turku, Finland; ^3^Department of Cognitive Neuroscience and Philosophy, University of Skövde, Skövde, Sweden; ^4^Department of Biomedical and Clinical Sciences, Linköping University, Linköping, Sweden

**Keywords:** emotion, emotion regulation, REM sleep, dreaming, continuity hypothesis

## Abstract

There is increasing evidence that sleep plays an important role in affective processing. However, it is unclear whether dreaming—the subjective experiences we have during sleep—also serves an affect regulation function. Here, we investigated the within-person relationship between negative affect experienced in dreams and next-day waking affect level, affect reactivity, and affect regulation. For 5 days, 40 participants reported their dreams and rated their dream affect and post-sleep waking affect level upon morning awakening. Thereafter, they performed an affect reactivity and regulation task which involved viewing neutral and negative pictures with the instruction either to simply view the pictures or to down-regulate the affect evoked by these pictures. Multilevel regression analyses showed that the more negative affect people experienced in their dreams at night, the more negative affect and the less positive affect they reported the next morning. However, negative dream affect was associated neither with affect reactivity to the pictures nor with the ability to down-regulate negative affect in response to these pictures. In fact, Bayesian analyses favored the null hypotheses. These findings fail to provide support for the affect regulation function of dreaming and, instead, speak for affective continuity between dreaming and post-sleep wakefulness.

## Introduction

Sleep, especially rapid eye movement (REM) sleep, plays an important role in affective processing. Poor sleep is a risk factor for a range of affective disorders, such as anxiety and depression (e.g., Alvaro et al., 2013). In non-clinical populations, sleep disturbances have been associated with next-day negative affect (Konjarski et al., 2018) and enhanced reactivity to affective stimuli (Altena et al., 2016), although evidence remains mixed (ten Brink et al., 2022). It has been argued that this occurs because sleep plays an important role in affect regulation and poor sleep impairs this process (Walker and van der Helm, 2009; Palmer and Alfano, 2017; Tempesta et al., 2018). However, it remains unclear whether the subjective experiences we have during sleep—our dreams—also contribute to affect regulation.

According to the so-called emotion regulation theories of dreaming, the function of dreams is to (re)process and regulate affect (Cartwright, 1991, 2010; Kramer, 1991, 1993; Hartmann, 1996, 2011; Levin and Nielsen, 2007; Perogamvros and Schwartz, 2012; Malinowski and Horton, 2015). Despite some variation in the specifics of these theories, they all agree that dreams incorporate and reprocess the memories of affective experiences of the waking life, integrate them with existing memory elements, to ultimately downregulate their intensity and thus help us cope better with these experiences during wakefulness. Affect in dreams either reflects this process or is a necessary condition for regulation to take place (Malinowski and Horton, 2015). Most of these theories attribute a special role to negative affect: dreams specifically incorporate negative affect (e.g., fear) and the processing of this leads to more adaptive responses to negative (e.g., threatening) stimuli in wakefulness, akin to fear extinction (Scarpelli et al., 2019). This applies to so-called normal dreams and occasional bad dreams, because frequent nightmares reflect a failure of the affect regulation function (Levin and Nielsen, 2007). Dreaming is thus seen as an “emotional thermostat” (Kramer, 1991, 1993) or “overnight therapy” (Hartmann, 1996; Walker and van der Helm, 2009) that aids affective adaptation in wakefulness. Yet, it is not entirely clear from these theories how exactly the affect regulation function is meant to be reflected in waking affect. Some of the possible predictions derived from these theories are that negative dream affect may lead to (a) a less negative, and more positive, post-sleep *affect level* upon morning awakening; (b) lower *affect reactivity* to (negative) stimuli in wakefulness; and (c) improved *affect regulation* as such.

In contrast to emotion regulation theories, the so-called continuity theories of dreaming^1^ assume that there is continuity between waking and dream experiences, that is, dream experiences do not serve any particular function but simply reflect waking events, experiences, and concerns (Schredl, 2003, 2018; Domhoff, 2017, 2018). Different types of continuity can be distinguished. For example, whereas thematic continuity refers to certain themes being continuous across wakefulness and dreaming (e.g., studying for exams in waking life and dreaming about exams), affective continuity refers to the affective tone of waking life events being continuous with dream affect, irrespective of the specific thematic content (e.g., watching a movie about zombies and having a nightmare about being late to the exam) (Schredl, 2018). According to affective continuity (the focus of this paper), pre-and post-sleep waking affect is continuous with dream affect, with the more affectively intense daytime events being more likely to be incorporated into dreams (Schredl and Hofmann, 2003; Schredl, 2018) and the affective nature of the dream, in turn, influencing affect experienced in subsequent waking life (Schredl and Reinhard, 2009–2010). Thus, negative dream affect reflects enhanced negativity in waking life (which could occur due to state and/or trait factors).

To date, most research has focused on the relationship between dream affect and (post-sleep) waking affect level. Whereas some earlier studies supported emotion regulation theories of dreaming, demonstrating that negatively valenced dreams are associated with more positively valenced post-sleep affect (e.g., Cohen and Cox, 1975) or better coping with adverse life experiences (e.g., Cartwright, 1991, 2010), more recent studies lend greater support for the continuity theories, reporting positive correlations between dream affect and post-sleep affect (e.g., Schredl and Doll, 1998; Yu, 2007; Mallett et al., 2021; Barbeau et al., 2022a). Importantly, studies directly testing the affect regulation function of dreaming have often failed to find evidence for the affect regulation function (e.g., De Koninck and Koulack, 1975; Tousignant et al., 2022).

Few studies have investigated the relationship between dream affect and affect reactivity in wakefulness. In one recent study, Sterpenich et al. (2019) found that individuals who tended to experience negative affect, especially fear, in their home dreams had decreased activity in affect-generative brain areas (i.e., amygdala, right insula) and increased activity in affect-regulatory brain areas (i.e., medial prefrontal cortex) in response to aversive stimuli in wakefulness. The authors concluded that experiencing negative affect in dreams (beyond sleep) is associated with more adaptive affect regulation in wakefulness. However, because the authors studied between-person variability of dream affect and its relationship to affect reactivity, it remains unknown how dream affect is linked to next-day affect reactivity and regulation within individuals. In another study, Lara-Carrasco et al. (2009) showed that participants who experienced less intense negative affect in laboratory REM sleep dreams displayed the highest evening-to-morning decreases in affect reactivity, as reflected in negativity ratings of pictures. These findings suggest that negative dream affect is not associated with decreased, but increased, affective reactivity in subsequent wakefulness and, therefore, provide support for the continuity theories of dreaming.

Thus, findings regarding the link between dream affect and post-sleep waking affect level and affect reactivity are mixed. Importantly, to date, no studies have directly examined the link between dream affect and waking affect regulation as such. In this study, we addressed this gap by investigating the within-person relationship between dream affect and next-day post-sleep waking affect level, affect reactivity, and affect regulation. Our focus on within- rather than between-person association was motivated by the goal of providing the most direct possible test of the emotion regulation vs. continuity theories of dreaming. We did so by directly opposing predictions derived from these theories (see also Revonsuo et al., 2016). Although both emotion regulation theories and continuity theories agree that pre-sleep waking affect influences dream affect in a corresponding manner, they differ in their predictions regarding the effect of dream affect on subsequent post-sleep affect. According to emotion regulation theories, after a night with high (vs. low) negative dream affect, participants should display less negative (and more positive) post-sleep affect level, lower affect reactivity, and improved affect regulation ability. In contrast, the continuity theories would predict the opposite: after a night with high (vs. low) negative dream affect, participants should display more negative (and less positive) post-sleep affect level, higher affect reactivity, and lower affect regulation ability. We focused specifically on negative (rather than positive) dream affect because the emotion regulation theories of dreaming attribute a special role to negative affect. Furthermore, since the emotion regulation theories argue that dreaming *per se*, beyond sleep, has an affect regulatory function, and due to the role of sleep in affective processing, we controlled for sleep quality in all the analyses.

## Materials and methods

### Participants

According to Arend and Schäfer (2019), two-level models that would yield sufficient power (≥0.80) to detect at least medium level-1 effect sizes, require sample sizes 30/5 (i.e., 5 measurement occasions from 30 participants) or 40/3 (i.e., 3 measurement occasions from 40 participants). Thus, we aimed to recruit 40 participants with at least 3 measurement occasions. To account for possible dropouts, and for the possibility of some participants reporting no dreams (or no affect experienced in dreams), we aimed to collect data from at least 50 participants.

Fifty-one healthy Finnish adults (44 females, 1 “other,” *M*_*age*_ = 25.18, SD_*age*_ = 7.12), who self-reported no neurological, psychiatric, or sleep disorders, and who were not on any medication affecting the central nervous system, participated in the study. Eleven participants were excluded during data preprocessing (see section “Data reduction”), leaving a final sample of 40 participants (33 females, 1 “other”) with an age range of 19–55 (*M* = 25.35, SD = 7.39) to be included in statistical analyses.

Participants were recruited *via* the University of Turku psychology students’ credit pool, mailing lists of Finnish universities, as well as *via* advertisements posted on social media. Participants did not receive any monetary compensation. However, psychology students at the University of Turku could receive course credits for their participation, and other participants had the opportunity to take part in a lottery (2 × 20 € gift cards) as compensation for their time.

### Experimental design and procedure

Participants first completed an online well-being questionnaire (administered *via* Webropol 3.0 survey tool). It contained demographic questions as well as scales measuring different aspects of well-being and ill-being, trait affect regulation, and general sleep quality. Since these data were collected in the framework of another study, these results will not be discussed further in this paper.

**FIGURE 1:**
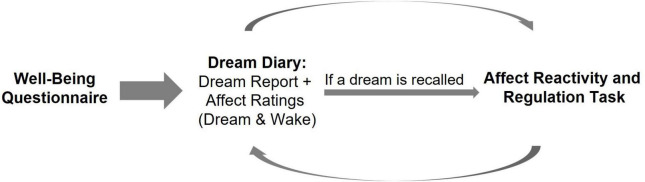
Procedure of the study. Participants first completed the well-being questionnaire. Thereafter, every morning upon awakening, they logged on to an online dream diary in which they answered questions about bed-time, waking time, sleep quality, and whether they remembered any dreams that night. If they recalled a dream, participants were asked to provide a narrative dream report and to rate the affect they experienced in the dream using dimensional and discrete affect rating scales. Participants also rated their post-sleep waking affect level. On the mornings when participants recalled a dream and provided dream affect ratings, they were instructed to carry out an affect reactivity and regulation task immediately upon filling in the dream diary. This procedure (i.e., filling in the dream diary and performing the task) was continued each morning until the participants had provided dream affect ratings and carried out the task five times (i.e., on five mornings).

After completing the online well-being questionnaire, participants kept an online home dream diary (*via* Webropol 3.0) until dream reports had been provided on five mornings (see Figure 1). Participants were instructed to fill in the diary each morning immediately upon awakening. The diary contained questions about bed-time the previous evening, awakening time for the morning of diary completion, sleep quality the previous night, and whether participants recalled having a dream last night. If a dream was remembered, participants were asked to report their dream(s) in as much detail as possible and to rate the affect they experienced in the dream (see section “Measures”). They were also asked to rate their momentary (i.e., post-sleep waking) affect using the same scale as for dream affect. On mornings when participants provided dream reports and ratings of dream affect, they were also instructed to carry out an affect reactivity and regulation task (see section “Measures”) immediately upon filling in the dream diary.

The study was conducted in line with the Declaration of Helsinki and was approved by the Ethical Committee for Human Sciences at the University of Turku, Finland. Informed consent was obtained from all participants prior to participation.

### Measures

#### Dream affect

Dream affect was measured using both dimensional and discrete rating scales. Using two unipolar dimensional scales, participants were asked to rate the extent to which they experienced positive affect (PA) and negative affect (NA) in the dream on a scale from 1 (not at all) to 5 (extremely).

For discrete affect, the modified Differential Emotions Scale (mDES; Fredrickson, 2013) was used. The scale has been shown to have good psychometric properties (Sikka et al., 2017; Conte et al., 2020) and the Finnish version of the scale has been used in previous studies investigating dream affect (Sikka et al., 2014, 2019). This 20-item scale measures 10 PA categories (e.g., “What is the most amused, fun-loving, or silly you felt?”) and 10 NA categories (e.g., “What is the most angry, irritated, or annoyed you felt?”) with three items per category. Participants were asked to think back to the dream they had had that night and to rate *the greatest amount* they experienced each of the affect items on a scale from 1 (not at all) to 5 (extremely). The 10 PA and 10 NA items were aggregated to form the PA (Cronbach’s *a* = 0.88) and NA (Cronbach’s *a* = 0.84) subscales, respectively.

For analyses, the mean scores of dimensional and discrete rating scales (i.e., mean of dimensional NA and discrete NA; mean of dimensional PA and discrete PA) were calculated separately for NA and PA for each dream.

#### Daily sleep quality

In the diary, participants were asked to rate the quality of their sleep during the preceding night on a scale from 1 (very good) to 4 (very bad). This item derives from the Pittsburgh Sleep Quality Index (Buysse et al., 1989).

#### Waking affect level

Waking affect level was measured using the same mDES scale as used to measure dream affect. Participants were asked to rate the extent to which they experienced each of the 20 affect items *in the present moment*. The 10 PA and 10 NA items were aggregated to form the PA (Cronbach’s *a* = 0.91) and NA (Cronbach’s *a* = 0.79) subscales, respectively.

#### Waking affect reactivity and regulation task

**FIGURE 2:**
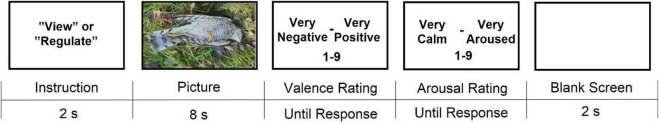
Timeline of a trial in the affect reactivity and regulation task. Valence and arousal ratings were accompanied by Self-Assessment Manikins (SAM; Bradley and Lang, 1994). The picture presented in this figure is shown for illustrative purposes only.

An online affect reactivity and regulation task was carried out *via* the Gorilla Experiment Builder platform^2^ (Anwyl-Irvine et al., 2020). The task (see Figure 2) was based on previous studies investigating the role of sleep in next-day affect reactivity and regulation (Reddy et al., 2017; Zhang et al., 2019; Shermohammed et al., 2020) and is widely used to manipulate affect reactivity and regulation. Participants were shown a set of affective (negative) and neutral pictures selected from the Nencki Affective Pictures System (NAPS; Marchewka et al., 2014). They were asked either (a) to *view* the picture, try to understand its content, and let themselves freely experience all the feelings it evokes (without trying to change what they were feeling in any way), or (b) to *regulate* (reappraise) the feelings elicited by the picture following previously given instructions. At the beginning of the task, participants were provided information regarding how to regulate their affect using reappraisal. Specifically, they were instructed to look carefully at the picture and try to re-interpret the meaning of the picture so that it would elicit less negative feelings in them. Participants were also provided different examples of how to down-regulate their negative feelings: to imagine that the situation depicted in the picture is not true, but part of a movie (“It’s just a movie”); to think that the situation depicted in the picture is getting better (“He will get better soon”); to think of a more positive explanation of the situation depicted in the picture (“Maybe he is tired, rather than lonely”); or to simply view the picture as a detached observer.

Every participant completed five sets of trials, each set performed on a separate day. The order of the sets was counterbalanced across participants. Each set consisted of 60 trials (20 view-neutral, 20 view-negative, and 20 regulate-negative) that were randomized within every set. The instruction (“view” or “regulate”) coupled with the negative pictures was randomized across participants so that each negative picture was shown to some participants with the instruction to “view” and to the others with the instruction to “regulate,” thus balancing any possible differences between the negative pictures used for each condition. Neutral pictures were always shown with the instruction to “view.” Before completing the first set of trials, participants completed a practice set consisting of one view-neutral, one view-negative, and one regulate-negative trial.

After having seen each picture for 8s, participants were asked to rate the valence and arousal they felt in response to it. These were rated on a 9-point Likert-type scale ranging from 1 (very negative/very calm) to 9 (very positive/very aroused) using the Self-Assessment Manikin (SAM; Bradley and Lang, 1994). A total of 300 NAPS pictures (200 negative, 100 neutral) were selected and divided into the five trial sets based on normative ratings (Marchewka et al., 2014; see Supplementary material).

Participants were allowed to carry out the task using either their computer or mobile phone, with the requirement to use the same device on all data collection days.

Affect reactivity was deemed higher when arousal ratings were higher and when valence ratings were lower (i.e., more negative) in response to viewing negative as compared to viewing neutral pictures. Affect regulation was evident when arousal ratings decreased and/or valence ratings increased (i.e., more positive) in response to the instruction to regulate one’s feelings when viewing negative pictures compared to when simply viewing negative pictures and freely experiencing the feelings these evoke.

### Data reduction

In total, 512 dream diaries were filled in by 51 participants (*M* = 10.04, SD = 5.85, range 2–28).^3^ In 69 (13.5%) of the dream diaries, participants reported having no dreams during the night and in 193 (37.7%) of the dream diaries, participants reported thinking they had a dream but not remembering it. A dream was reported and dream affect ratings provided in 250 (48.8%) of the dream diaries (*M* = 4.90, SD = 2.10, range 1–12).

Given that the memory of dream experiences is fleeting and subject to interference, we excluded days on which the dream diary was filled in after more than a 2 h delay between awakening and submitting the dream diary (*n* = 13). To ensure that the affect reactivity and regulation task would be carried out as close as possible to dream experiences, we also excluded days on which the task was performed more than 30 min after submitting the dream diary (*n* = 20). We also excluded days on which (a) there was a delay in submitting both the dream diary and performing the task (*n* = 5), (b) the participant carried out the task before filling in the dream diary (*n* = 2), (c) the participant only submitted the dream diary but failed to perform the task (*n* = 8), (d) there was an incorrect dream diary entry (*n* = 4). After excluding these days, we excluded participants who had dream reports from less than 3 days (*N*_*participants*_ = 11, *n*_*reports*_ = 18). Additionally, we excluded a day on which a participant performed the task but did not submit a dream diary (*n* = 1).

As a result of data reduction, 40 participants and 180 dream reports (*M* = 4.50, SD = 1.34, range 3–11 reports) were included in the analyses. Considering that three participants provided more than one report per day, the final data includes, on average, 4.28 (SD = 0.75, range 3–5) dreams and tasks from 171 days (i.e., days with both dream report and task).

### Data analyses

All analyses were carried out using IBM SPSS Statistics (v. 20) and R (v. 4.0.2, R Core Team, 2020). We performed linear mixed-effects regression models (also known as multilevel or hierarchical models; Hox, 2010) using the function *lme*r from the packages *lme4* (Bates et al., 2015) and *lmerTest* (Kuznetsova et al., 2017). These models account for the nested nature of the data, i.e., several dream affect ratings and task results per participant. The Bayesian version of the linear mixed-effects model was performed using the *brms* package (Bürkner, 2017), which is based on *Stan* (Carpenter et al., 2017). The Bayes Factors (*BF*) for model comparisons were estimated based on the bridge sampling method (Bürkner, 2017).

To test the relationship between dream affect and post-sleep waking affect level, post-sleep positive and negative affect were specified as outcome variables, whereas dream NA (mean of dimensional and discrete ratings) was included as a predictor. To test task manipulation effectiveness, task valence and arousal ratings were specified as outcome variables, whereas condition (0 = view-neutral; 1 = view-negative; 2 = regulate-negative; contrast-coded) was included as a predictor. To test the relationship between dream NA and task performance, we additionally included dream NA as well as condition *dream NA interaction as predictors. In all models we controlled for age, gender (0 = male, 1 = female, 2 = other), daily sleep quality, and the device (0 = computer; 1 = mobile phone) used to carry out the task. Participant-specific random intercept was also included in all the models (to account for the nested data). All Level-1 predictors (dream NA, daily sleep quality) were group-mean centered because this removes between-participant variation from the predictors and gives a “pure” estimate of the within-participant regression coefficient (Enders and Tofighi, 2007; Nezlek, 2012). Continuous level-2 variables (age) were grand-mean centered.

We used the *anova* function to compare different models. We also calculated marginal and conditional *R*^2^ using the *r.squaredGLMM* function in *MuMIN* package (Barton, 2022). Whereas the marginal *R*^2^ represents the variance explained by the fixed effects, the conditional *R*^2^ represents the variance explained by the whole model, including both fixed and random effects (Nakagawa and Schielzeth, 2013).

We also repeated all the analyses using dream PA (mean of dimensional and discrete ratings) as a predictor. These secondary analyses yielded similar results as with dream NA (see Supplementary material).

Results were considered significant if *p* < 0.05. For non-significant results, a follow-up Bayesian analysis was conducted and *BF* < 1/3 indicated substantial evidence in favor of the null hypothesis H_0_ (Wetzels and Wagenmakers, 2012).

## Results

### Dream affect and post-sleep waking affect level

**FIGURE 3:**
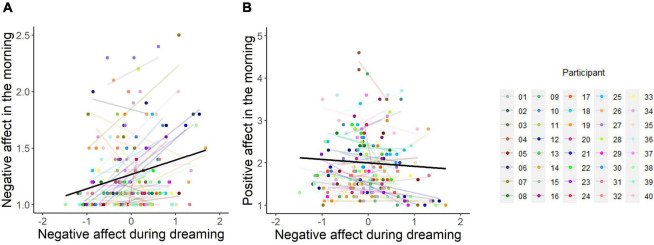
Relationship between negative affect experienced in dreams and subjective ratings of post-sleep negative affect **(A)** and positive affect **(B)** the following morning. Figures are displayed for visualization purposes only since analyses were based on linear mixed-effects models with covariates. The black fitted line indicates model prediction, whereas each colored line represents a regression line for each individual participant. Dots of the same color indicate repeated measurements within a participant. Negative affect during dreaming has been group-mean centered, reflecting variation around each participant’s own mean values. The y-axis on panel A has been truncated to better visualize individual regression lines.

According to emotion regulation theories, dream NA should predict less negative (and more positive) post-sleep affect level, whereas the continuity theories would predict more negative (and less positive) post-sleep affect level. Linear mixed-effects regression models showed that dream NA was positively associated with negative affect in the morning (*B* = 0.129, 95% CI [0.098; 0.160], SE = 0.016, *t* = 8.121, *p* < 0.001, marginal *R*^2^ = 0.073, conditional *R*^2^ = 0.609) but negatively associated with positive affect in the morning (*B* = −0.083, 95% CI [−0.140; −0.026], SE = 0.029, *t* = −2.839, *p* < 0.01, marginal *R*^2^ = 0.128, conditional *R*^2^ = 0.711) (see Figure 3). Similar results were obtained with dream PA as a predictor (see secondary analyses in Supplementary material).

Additionally, sleep quality during the night was a significant predictor of morning affect, with nights rated to have lower sleep quality associated with lower positive affect upon awakening (*B* = −0.181, 95% CI [−0.253; −0.109], SE = 0.037, *t* = −4.922, *p* < 0.001). No significant relationships occurred between sleep quality and negative affect in the morning (*B* = 0.034, 95% CI [−0.005; 0.073], SE = 0.020, *t* = 1.688, *p* = 0.092).

### Dream affect and post-sleep waking affect reactivity and regulation

First, we tested the effectiveness of task manipulation. As expected, condition was a significant predictor of task valence. Specifically, viewing negative pictures was associated with lower valence (i.e., more negative) ratings as compared to viewing neutral pictures (*B* = −1.731, 95% CI [−1.846; −1.616], SE = 0.059, *t* = −29.572, *p* < 0.001) (see Supplementary Figure 1). Regulating negative pictures was associated with higher valence (i.e., more positive) ratings as compared to viewing negative pictures (*B* = 0.739, 95% CI [0.624; 0.854], SE = 0.059, *t* = 12.625, *p* < 0.001) but with lower valence ratings when compared to viewing neutral pictures (*B* = −0.992, 95% CI [−1.107; −0.877], SE = 0.059, *t* = −16.947, *p* < 0.001). Similar results were obtained for task arousal ratings. Viewing negative pictures was associated with higher arousal ratings as compared to viewing neutral pictures (*B* = 1.406, 95% CI [1.286; 1.527], SE = 0.061, *t* = 22.983, *p* < 0.001). Regulating negative pictures was associated with lower arousal ratings as compared to viewing negative pictures (*B* = −0.444, 95% CI [−0.564; −0.323], SE = 0.061, *t* = −7.249, *p* < 0.001) but with higher arousal ratings when compared to viewing neutral pictures (*B* = 0.963, 95% CI [0.843; 1.083], SE = 0.061, *t* = 15.734, *p* < 0.001). Together, these results indicate that task manipulation was effective: participants’ affective reactivity was higher when viewing negative (as compared to viewing neutral) pictures, and they were successful in regulating their affect in response to negative pictures when instructed to do so. However, as indicated by the significant difference between the regulate-negative and view-neutral conditions for both valence and arousal, participants did not manage to fully “neutralize” their affective reactions to negative pictures.

Second, we tested the relationship between dream affect and waking affect reactivity and regulation. According to emotion regulation theories, higher levels of dream NA should predict lower next-day affect reactivity and improved affect regulation ability, whereas the continuity theories would predict higher next-day affect reactivity and lower affect regulation ability. However, results yielded no significant effects of dream NA (valence: *B* = −0.100, 95% CI [−0.236; 0.035], SE = 0.069, *t* = −1.454, *p* = 0.147; arousal: *B* = 0.099, 95% CI [−0.042; 0.241], SE = 0.072, *t* = 1.381, *p* = 0.168) nor condition*dream NA interactions (valence: *F*(2, 499) = 0.792, *p* = 0.454; arousal: *F*(2, 500) = 0.372, *p* = 0.690) on either task valence or arousal ratings. Models including condition*dream NA interactions (valence: marginal *R*^2^ = 0.594, conditional *R*^2^ = 0.645; arousal: marginal *R*^2^ = 0.268, conditional *R*^2^ = 0.819) were not significantly different from models including condition as the main effect only (valence: marginal *R*^2^ = 0.592, conditional *R*^2^ = 0.643; arousal: marginal *R*^2^ = 0.266, conditional *R*^2^ = 0.818), χ^2^(3) = 2.926, *p* = 0.403 (valence), χ^2^(3) = 2.110, *p* = 0.550 (arousal).

These null effects were confirmed in the Bayesian version of the models (valence: *BF* = 0.022; arousal: *BF* = 0.017).

## Discussion

We investigated the within-person relationship between dream affect and next-day affect level, affect reactivity, and affect regulation. Results showed that dream affect was associated with affect level the next morning: participants who experienced higher levels of NA (or lower levels of PA) in their dreams exhibited more negative and less positive post-sleep affect the next morning. These findings corroborate previous studies demonstrating a positive association between dream affect and post-sleep waking affect (Yu, 2007; Schredl and Reinhard, 2009–2010; Mallett et al., 2021). However, hypotheses regarding the relationship between dream NA and next-day affect reactivity and affect regulation were not supported. Although negative pictures induced higher affect reactivity (as reflected in higher arousal and more negative ratings of the pictures) and participants were successful in down-regulating negative affect when instructed to do so, neither affect reactivity nor regulation were associated with dream NA. In fact, Bayesian analyses provided support in favor of the null hypotheses, that is, no relationship between dream NA and waking affect reactivity or regulation.

The findings of the present study fail to provide support for the emotion regulation theories of dreaming, which argue that experiencing NA in dreams contributes to affect regulation in subsequent wakefulness. Instead, results are more in line with the continuity theories of dreaming and suggest affective continuity between dream affect and post-sleep waking affect level. Differences between results regarding self-reported affect level upon awakening versus affect reactivity to stimuli indicates that dream affect is more associated (or continuous) with naturally occurring affect, rather than experimentally manipulated affect. However, it cannot be ruled out that significant correlations between negative dream affect and self-reported post-sleep affect reflect a simple carry-over effect with the physiological arousal evoked by dream affect continuing into wakefulness (Schredl, 2009).

Although the present study did not provide support for the role of dream affect in waking affect reactivity and regulation at the within-person level, it is possible that this relationship exists at the between-individual level. Sterpenich et al. (2019) showed that individuals who tend to experience more NA in dreams display reduced reactivity to affective pictures in wakefulness. Hence, dream affect may be more likely linked to individual differences in habitual affect reactivity and affect regulation (i.e., trait affect reactivity and regulation). This may be especially apparent in those characterized by maladaptive affect regulation (Levin and Nielsen, 2007). Future studies (with appropriate power to evaluate individual differences) investigating the relationship between dream affect and trait affect reactivity and regulation are needed to test this proposition.

Another reason for null findings with regard to affect regulation may be that, in the present study, affect reactivity and regulation were investigated using subjective ratings of pictures. Previous studies have shown altered brain responses to affective stimuli following sleep loss (Zhang et al., 2019), and in relation to experiencing negative dream affect (Sterpenich et al., 2019), even in the absence of differences in subjective ratings (Zhang et al., 2019). Given the relatively low coherence between the subjective experience and physiological components of affective experiences (Mauss et al., 2005), it is possible that different results would be obtained using physiological measures of affective reactivity and regulation.

It is also possible that waking affect regulation is not associated with dream affect in general but with those affective experiences in dreams that are related to the processing of particularly salient memories of real-life experiences (Malinowski and Horton, 2015). Similarly, it is likely that the affect regulation function is only apparent when individuals experience a certain level of stress during the day that then activates the need for regulation (Levin and Nielsen, 2007; Barbeau et al., 2022b). Although the findings regarding the relationship between pre-sleep affect and dream affect are mixed (e.g., Koulack et al., 1985; Gilchrist et al., 2007; Yu, 2007; cf. Samson-Daoust et al., 2019; Sikka et al., 2019; Conte et al., 2020), accumulated stress over a longer period of time may influence dream affect and, *via* the activation of regulation processes, morning affect. Additionally, affect regulation may not occur within one night, but may be a longer-term process occurring across several nights (akin to the “dream lag” effect; Blagrove et al., 2011), and perhaps even weeks or months (Cartwright, 2010; Goldstein and Walker, 2014). Thus, future studies should strive to manipulate pre-sleep affect, measure both short-term (on the day prior to sleep) and longer-term (days or weeks prior to sleep) stress in the waking life, investigate how dream affect is related to particularly important waking life events that have been incorporated into dreams, and collect data over a longer time period.

The findings of the current study should be considered in light of several limitations. First, since participants rated their waking affect right after rating their dream affect, it is possible that ratings of waking affect were biased by dream affect ratings. While it is not possible to obtain affect ratings while the dream is ongoing, the order of rating dream and waking affect should be counterbalanced in future studies, albeit waking affect ratings may interfere with the dream memory.

Second, we did not measure pre-sleep waking affect. It is likely that affect the next morning would be explained more by pre-sleep waking affect rather than dream affect (Barbeau et al., 2022a), although there is also evidence that the effect of previous-day events on next-day affect occurs *via* dream affect (Schredl and Reinhard, 2009–2010). To better understand the extent to which such cross-state affective continuity depends on the affective nature of dreams, it is important to measure, and control for, pre-sleep affect in future studies.

Third, the fact that this was a home dream study, and data was collected online, made it difficult to control for the exact time when participants filled in the dream diary and carried out the affect reactivity/regulation task. A temporal lag between the actual dream experience and ratings of dream affect may introduce memory biases (e.g., Sikka, 2019). Similarly, a temporal lag between the dream experience and task means that waking events occurring during this lag may have influenced task performance. Although, we tried to control for temporal lags by removing dream diaries filled in too long following awakening and tasks carried out too long after filling in the dream diary, future studies could benefit from tighter experimental control. Relatedly, it was not possible to monitor how well participants followed instructions, especially those pertaining to affect regulation. In future studies, it would be beneficial to obtain participants’ evaluations of their regulation success as one indication of task performance.

Fourth, it is also possible that the data collection environment may have influenced the results. Previous studies have demonstrated that differences in the affective content of dreams depend on whether data have been collected in the home or laboratory setting (Sikka et al., 2018). Despite being ecologically more valid, the fact that sleep was not monitored at home means that it was not possible to control for the sleep stage as well as the time of the night from which the dreams (and related affect) derived. The fact that the affect regulation function is specifically postulated to apply to REM sleep, may be one reason for not finding any evidence in support of emotion regulation function of dreaming. Thus, future studies should replicate this study in a laboratory environment as well as in a home environment using sleep monitoring devices.

Finally, our results only pertain to dreams that participants were able to remember and report. This issue is common to almost all dream research since we do not have access to forgotten dreams. If dreams have an affect regulation function, this function should be operative irrespective of whether the dreams are remembered or not. Yet, it may be hypothesized that dream recall is higher when the affect regulation function fails, as in the case of nightmares (Levin and Nielsen, 2007). As a result, we may have access to a biased sample of dreams—those in which the affect regulation function is malfunctioning. However, this argument is not supported in the present study because only 9 of the 180 dreams (i.e., 5%) were rated as nightmares by participants, indicating that the majority of the dreams were so-called normal dreams. Nevertheless, dream recall is influenced by several trait and state variables (e.g., Schredl, 2018), all of which highlights the need to control for these potential factors.

In summary, the findings of the present study fail to provide support for the affect regulation function of dreaming and, instead, speak for affective continuity between dreaming and post-sleep wakefulness.

## Data availability statement

The original data set and analysis script can be found in a publicly accessible repository: https://osf.io/hsymd/.

## Ethics statement

The studies involving human participants were reviewed and approved by Ethical Committee for Human Sciences at the University of Turku, Finland. The patients/participants provided their written informed consent to participate in this study.

## Author contributions

PS conceptualized and designed the study and wrote the first manuscript. JG contributed to the study conceptualization. HE contributed to writing the first manuscript. PS and HE collected the data. PS and JZ analyzed the data. All authors reviewed and edited the manuscript.

## Footnotes

1 Varying conceptualizations of the Continuity Hypothesis exist (Domhoff, 1996, 2017; Schredl, 2003, 2017). For clarity, we refer to these here as continuity theories of dreaming.

2
www.gorilla.sc

3 Dream diaries were filled in each morning even when no dreams were recalled. Hence, most participants provided more than five diaries since the requirement was to fill in the diaries until there were dream reports and dream affect ratings from 5 days.
