# Supplementary material for: Negative dream affect is associated with next-day affect level, but not with affect reactivity or affect regulation
Source: Front Behav Neurosci. 2022 Oct 19;16:981289. doi: 10.3389/fnbeh.2022.981289 (PMC9626956; doi:10.3389/fnbeh.2022.981289)
Supplement: Supplementary file 1 [file Data_Sheet_1.docx]

**Supplementary Materials**

**Normative Ratings of Pictures Used in the Affect Reactivity and Regulation Task**

The NAPS pictures (see list of used pictures below) were selected for the five sets of trials based on the normative ratings from previous studies (Marchewka et al., 2014). Neutral pictures used for the different sets of trials did not significantly differ from each other in their normative ratings of valence (*F*(4, 95) = 1.668, *p* = .164) or arousal (*F*(4, 95) = 0.356, *p* = .839). Similarly, there were no significant differences between the normative valence (*F*(4, 195) = 0.361, *p* = .836) and arousal (*F*(4, 195) = 0.573, *p* = .682) ratings of negative pictures used for the five sets of trials. Furthermore, there were no significant differences in normative valence and arousal ratings between the pictures used for the “view” vs “regulate” conditions for each of the five sets of trials (*p* > 0.05). Importantly, the normative ratings of negative pictures were lower (*t*(277,69) = -57.94, *p* < 0.001) and arousal ratings higher (*t*(236.15) = 25.23, *p* < 0.001) than neutral pictures, i.e., negative pictures were rated as more negative and more arousing than neutral pictures.

**Table S1**

*Labels of Pictures Used in the Task (from the NAPS database)*

| **Neutral pictures** | **Negative pictures** | **Negative pictures** |
| --- | --- | --- |
| Animals_127_h | Animals_074_h | Objects_039_h |
| Animals_003_h | Animals_001_h | Objects_088_h |
| Animals_005_h | Animals_012_h | Objects_109_h |
| Animals_026_h | Animals_013_h | Objects_111_h |
| Animals_043_h | Animals_016_h | Objects_117_h |
| Animals_044_h | Animals_018_h | Objects_120_h |
| Animals_080_h | Animals_019_h | Objects_125_h |
| Animals_082_h | Animals_024_h | Objects_126_h |
| Animals_083_h | Animals_025_h | Objects_132_h |
| Animals_088_h | Animals_027_h | Objects_139_h |
| Animals_093_h | Animals_032_h | Objects_141_h |
| Animals_105_h | Animals_033_h | Objects_144_h |
| Animals_129_h | Animals_037_h | Objects_145_h |
| Animals_138_h | Animals_038_h | Objects_148_h |
| Animals_148_h | Animals_039_h | Objects_149_h |
| Faces_026_h | Animals_041_h | Objects_153_h |
| Faces_030_h | Animals_042_h | Objects_154_h |
| Faces_055_h | Animals_045_h | Objects_157_h |
| Faces_060_h | Animals_048_h | Objects_158_h |
| Faces_180_h | Animals_050_h | Objects_218_h |
| Faces_192_h | Animals_053_h | Objects_232_h |
| Faces_194_h | Animals_054_h | Objects_233_h |
| Faces_200_h | Animals_056_h | Objects_243_h |
| Faces_208_h | Animals_057_h | Objects_283_h |
| Faces_211_h | Animals_060_h | Objects_285_h |
| Faces_216_h | Animals_062_h | People_001_h |
| Faces_218_h | Animals_063_h | People_003_h |
| Faces_264_h | Animals_067_h | People_004_h |
| Faces_276_h | Animals_068_h | People_006_h |
| Faces_286_h | Animals_071_h | People_007_h |
| Faces_292_h | Animals_075_h | People_008_h |
| Faces_309_h | Animals_077_h | People_009_h |
| Faces_311_h | Animals_078_h | People_010_h |
| Faces_312_h | Animals_207_h | People_012_h |
| Faces_336_h | Animals_221_h | People_016_h |
| Landscapes_016_h | Faces_003_h | People_017_h |
| Landscapes_019_h | Faces_007_h | People_020_h |
| Landscapes_036_h | Faces_009_h | People_021_h |
| Landscapes_044_h | Faces_010_h | People_022_h |
| Landscapes_057_h | Faces_011_h | People_023_h |
| Landscapes_060_h | Faces_013_h | People_038_h |
| Landscapes_063_h | Faces_016_h | People_058_h |
| Landscapes_067_h | Faces_018_h | People_071_h |
| Landscapes_076_h | Faces_019_h | People_082_h |
| Landscapes_080_h | Faces_028_h | People_083_h |
| Landscapes_081_h | Faces_032_h | People_084_h |
| Landscapes_091_h | Faces_034_h | People_085_h |
| Landscapes_093_h | Faces_041_h | People_086_h |
| Landscapes_111_h | Faces_146_h | People_087_h |
| Landscapes_135_h | Faces_150_h | People_094_h |
| Objects_008_h | Faces_152_h | People_098_h |
| Objects_014_h | Faces_158_h | People_118_h |
| Objects_046_h | Faces_159_h | People_119_h |
| Objects_049_h | Faces_164_h | People_120_h |
| Objects_050_h | Faces_170_h | People_121_h |
| Objects_059_h | Faces_172_h | People_123_h |
| Objects_065_h | Faces_174_h | People_124_h |
| Objects_067_h | Faces_176_h | People_125_h |
| Objects_089_h | Faces_270_h | People_126_h |
| Objects_119_h | Faces_271_h | People_127_h |
| Objects_130_h | Faces_274_h | People_128_h |
| Objects_160_h | Faces_283_h | People_129_h |
| Objects_162_h | Faces_284_h | People_133_h |
| Objects_185_h | Faces_285_h | People_136_h |
| Objects_189_h | Faces_287_h | People_140_h |
| Objects_196_h | Faces_288_h | People_142_h |
| Objects_204_h | Faces_290_h | People_143_h |
| Objects_208_h | Faces_291_h | People_144_h |
| Objects_211_h | Faces_293_h | People_145_h |
| Objects_221_h | Faces_294_h | People_147_h |
| Objects_223_h | Faces_298_h | People_197_h |
| Objects_230_h | Faces_300_h | People_198_h |
| Objects_231_h | Faces_302_h | People_200_h |
| Objects_237_h | Faces_303_h | People_202_h |
| Objects_244_h | Faces_366_h | People_206_h |
| Objects_245_h | Faces_367_h | People_208_h |
| Objects_247_h | Faces_368_h | People_214_h |
| Objects_269_h | Landscapes_001_h | People_215_h |
| Objects_274_h | Landscapes_002_h | People_216_h |
| Objects_276_h | Landscapes_004_h | People_217_h |
| Objects_278_h | Landscapes_005_h | People_219_h |
| Objects_279_h | Landscapes_007_h | People_220_h |
| Objects_296_h | Landscapes_010_h | People_221_h |
| Objects_299_h | Landscapes_011_h | People_222_h |
| Objects_306_h | Landscapes_014_h | People_223_h |
| Objects_308_h | Landscapes_017_h | People_224_h |
| Objects_310_h | Landscapes_022_h | People_225_h |
| Objects_311_h | Landscapes_026_h | People_226_h |
| Objects_312_h | Landscapes_068_h | People_227_h |
| Objects_313_h | Landscapes_139_h | People_230_h |
| People_056_h | Landscapes_177_h | People_232_h |
| People_091_h | Objects_001_h | People_233_h |
| People_095_h | Objects_002_h | People_235_h |
| People_097_h | Objects_003_h | People_237_h |
| People_104_h | Objects_004_h | People_238_h |
| People_146_h | Objects_006_h | People_239_h |
| People_149_h | Objects_007_h | People_240_h |
| People_150_h | Objects_011_h | People_241_h |
| People_155_h | Objects_013_h | People_243_h |
| People_159_h | Objects_022_h | People_246_h |

**Figure S1**

*Arousal (a) and Valence (b) Ratings in the Affect Reactivity and Regulation Task Across Different Conditions*

**
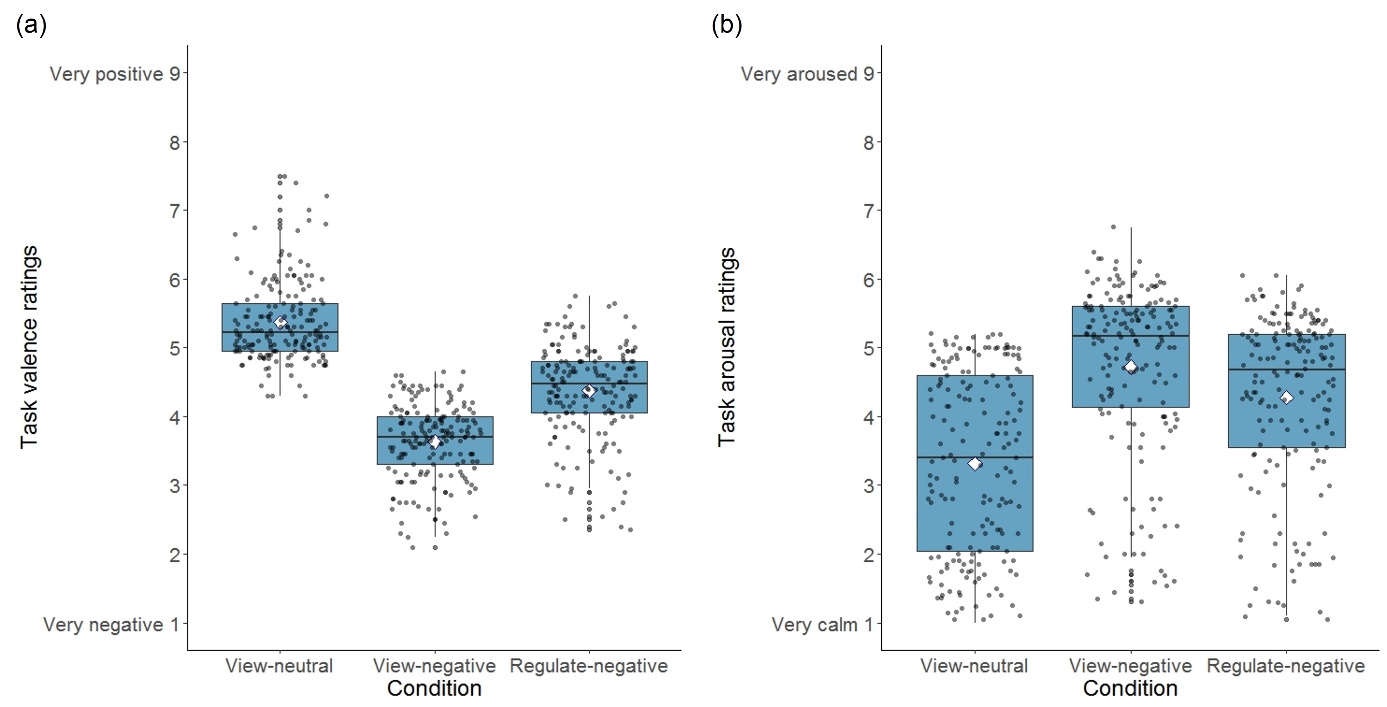
**

*Note*. Boxplots are based on 171 measurement occasions (i.e., days when the task was carried out) of 40 participants. ◊ = mean; horizontal line = median; box = 25th and 75th percentiles; whiskers = minimum and maximum values.

**Secondary Analyses**

We repeated all the analyses using dream PA (mean of discrete and dimensional PA) as a predictor. Regarding post-sleep affect level in the morning, dream PA was positively associated with positive affect in the morning (*B* = 0.273, 95% CI [0.221; 0.324], *SE* = 0.026, *t* = 10.347, *p* < .001, marginal *R^2^* = 0.173, conditional *R^2^* = 0.759) but negatively associated with negative affect in the morning (*B* = -0.109, 95% CI [-0.140; -0.078], *SE* = 0.016, *t* = -6.849, *p* < .001, marginal *R^2^* = 0.052, conditional *R^2^* = 0.592).

Regarding waking affect reactivity and regulation, as with dream NA, there were no significant effects of dream PA (valence: *B* = 0.068, 95% CI [-0.063; 0.199], *SE* = 0.067, *t* = 1.017, *p* = .310; arousal: *B* = -0.004, 95% CI [-0.142; 0.134], *SE* = 0.070, *t* = -0.060, *p* = .952) nor condition-dream PA interactions (valence: *F*(2, 499) = 1.531, *p* = .217; arousal: *F*(2, 500) = 0.103, *p* = .902). These null effects were confirmed in the Bayesian version of the models (valence: BF = 0.022; arousal: BF = 0.006).
